# Publisher Correction: Reproducible brain-wide association studies require thousands of individuals

**DOI:** 10.1038/s41586-022-04692-3

**Published:** 2022-05-09

**Authors:** Scott Marek, Brenden Tervo-Clemmens, Finnegan J. Calabro, David F. Montez, Benjamin P. Kay, Alexander S. Hatoum, Meghan Rose Donohue, William Foran, Ryland L. Miller, Timothy J. Hendrickson, Stephen M. Malone, Sridhar Kandala, Eric Feczko, Oscar Miranda-Dominguez, Alice M. Graham, Eric A. Earl, Anders J. Perrone, Michaela Cordova, Olivia Doyle, Lucille A. Moore, Gregory M. Conan, Johnny Uriarte, Kathy Snider, Benjamin J. Lynch, James C. Wilgenbusch, Thomas Pengo, Angela Tam, Jianzhong Chen, Dillan J. Newbold, Annie Zheng, Nicole A. Seider, Andrew N. Van, Athanasia Metoki, Roselyne J. Chauvin, Timothy O. Laumann, Deanna J. Greene, Steven E. Petersen, Hugh Garavan, Wesley K. Thompson, Thomas E. Nichols, B. T. Thomas Yeo, Deanna M. Barch, Beatriz Luna, Damien A. Fair, Nico U. F. Dosenbach

**Affiliations:** 1 Department of Psychiatry, Washington University School of Medicine, St Louis, MO USA; 2 Department of Psychiatry, Massachusetts General Hospital, Harvard Medical School, Boston, MA USA; 3 Department of Psychology, University of Pittsburgh, Pittsburgh, PA USA; 4 Department of Psychiatry, University of Pittsburgh, Pittsburgh, PA USA; 5 Department of Bioengineering, University of Pittsburgh, Pittsburgh, PA USA; 6 Department of Neurology, Washington University School of Medicine, St Louis, MO USA; 7 University of Minnesota Informatics Institute, University of Minnesota, Minneapolis, MN USA; 8 Department of Psychology, University of Minnesota, Minneapolis, MN USA; 9 Masonic Institute for the Developing Brain, University of Minnesota Medical School, Minneapolis, MN USA; 10 Department of Pediatrics, University of Minnesota Medical School, Minneapolis, MN USA; 11 Department of Psychiatry, Oregon Health and Science University, Portland, OR USA; 12 Minnesota Supercomputing Institute, University of Minnesota, Minneapolis, MN USA; 13 Department of Electrical and Computer Engineering, National University of Singapore, Singapore, Singapore; 14 Centre for Sleep and Cognition, National University of Singapore, Singapore, Singapore; 15 Centre for Translational MR Research, National University of Singapore, Singapore, Singapore; 16 N.1 Institute for Health, Institute for Digital Medicine, National University of Singapore, Singapore, Singapore; 17 Department of Biomedical Engineering, Washington University in St Louis, St Louis, MO USA; 18 Department of Cognitive Science, University of California San Diego, La Jolla, CA USA; 19 Department of Radiology, Washington University School of Medicine, St Louis, MO USA; 20 Department of Neurological Surgery, Washington University School of Medicine, St Louis, MO USA; 21 Department of Psychological and Brain Sciences, Washington University in St Louis, St Louis, MO USA; 22 Department of Psychiatry, University of Vermont, Burlington, VT USA; 23 Division of Biostatistics, University of California San Diego, La Jolla, CA USA; 24 Oxford Big Data Institute, Li Ka Shing Centre for Health Information and Discovery, Nuffield Department of Population Health, University of Oxford, Oxford, UK; 25 Integrative Sciences and Engineering Programme, National University of Singapore, Singapore, Singapore; 26 Martinos Center for Biomedical Imaging, Massachusetts General Hospital, Charlestown, MA USA; 27 Institute of Child Development, University of Minnesota Medical School, Minneapolis, MN USA; 28 Program in Occupational Therapy, Washington University School of Medicine, St Louis, MO USA; 29 Department of Pediatrics, Washington University School of Medicine, St Louis, MO USA

**Keywords:** Cognitive neuroscience, Psychology

Correction to: *Nature* 10.1038/s41586-022-04492-9 Published online 16 March 2022

Due to a tracking error during the peer review process, this article was published with the wrong received date. This article was received 19 May 2021, not 20 August 2020. Further, this article was originally published under the standard Springer Nature license (© The Author(s), under exclusive licence to Springer Nature Limited). It is now available as an open-access paper under a Creative Commons Attribution 4.0 International license, © The Author(s). The error has been corrected in the online version of the article

