# Supplementary material for: Reproducible brain-wide association studies require thousands of individuals
Source: Nature. 2022 Mar 16;603(7902):654–60. doi: 10.1038/s41586-022-04492-9 (PMC8991999; doi:10.1038/s41586-022-04492-9)
Supplement: Supplementary file 1 — The file contains Supplementary Discussion, Supplementary Figs. 1–17 and Supplementary Tables 1–4. [file 41586_2022_4492_MOESM1_ESM.pdf]

nature portfolio

https://doi.org/10.1038/s41586-022-04492-9

Supplementary information

Reproducible brain-wide association studies require thousands of individuals

In the format provided by the authors and unedited

Supplementary Information

Reproducible brain-wide association studies require thousands of individuals

Authors: Scott Marek[1,*Ω], Brenden Tervo-Clemmens[2,3*Ω], Finnegan J. Calabro[4,5], David F. Montez[6], Benjamin P. Kay[6], Alexander S. Hatoum[1], Meghan Rose Donohue[1], William Foran[4], Ryland L. Miller[1,6], Timothy J. Hendrickson[7], Stephen M. Malone[8], Sridhar Kandala[1], Eric Feczko[9,10], Oscar Miranda-Dominguez[9,10], Alice M. Graham[11], Eric A. Earl[9,11], Anders J. Perrone[9,11], Michaela Cordova[11], Olivia Doyle[11], Lucille A. Moore[11], Gregory M. Conan[9,11], Johnny Uriarte[11], Kathy Snider[11], Benjamin J. Lynch[9,12], James C. Wilgenbusch[9,12], Thomas Pengo[7,], Angela Tam[13-16], Jianzhong Chen[13-16], Dillan J. Newbold[6], Annie Zheng[6], Nicole A. Seider[6], Andrew N. Van[6,17], Athanasia Metoki[6], Roselyne J. Chauvin[6], Timothy O. Laumann[1], Deanna J. Greene[18], Steven E. Petersen[6,17,19-21], Hugh Garavan[22], Wesley K. Thompson[23], Thomas E. Nichols[24], B.T. Thomas Yeo[13-16,25,26], Deanna M. Barch[1,21], Beatriz Luna[3,4], Damien A. Fair[9,10,27,**Ω], Nico U.F. Dosenbach[6,17,19,28,29**Ω]

Affiliations:

[1]Department of Psychiatry, Washington University School of Medicine, St. Louis, MO 63110, USA
[2]Department of Psychiatry, Massachusetts General Hospital, Harvard Medical School, Boston, MA 02114, USA
[3]Department of Psychology, University of Pittsburgh, Pittsburgh, PA 15213, USA
[4]Department of Psychiatry, University of Pittsburgh, Pittsburgh, PA 15213, USA
[5]Department of Bioengineering, University of Pittsburgh, Pittsburgh, PA 15213, USA
[6]Department of Neurology, Washington University School of Medicine, St. Louis, MO 63110, USA
[7]University of Minnesota Informatics Institute, University of Minnesota, Minneapolis, MN 55455, USA
[8]Department of Psychology, University of Minnesota, Minneapolis, MN 55455, USA
[9]Masonic Institute for the Developing Brain, University of Minnesota Medical School, Minneapolis, MN 55455, USA
[10]Department of Pediatrics, University of Minnesota Medical School, Minneapolis, MN 55455, USA
[11]Department of Psychiatry, Oregon Health and Science University, Portland, OR 97239, USA
[12]Minnesota Supercomputing Institute, University of Minnesota, Minneapolis, MN 55455, USA
[13]Department of Electrical and Computer Engineering, National University of Singapore, Singapore 119077
[14]Centre for Sleep and Cognition, National University of Singapore, Singapore 119077

[15]Clinical Imaging Research Center, National University of Singapore, Singapore 119077
[16]N.1 Institute for Health, Institute for Digital Medicine, National University of Singapore, Singapore 119077
[17]Department of Biomedical Engineering, Washington University in St. Louis, St. Louis, MO 63130, USA
[18]Department of Cognitive Science, University of California San Diego, La Jolla, CA 92093, USA
[19]Department of Radiology, Washington University School of Medicine, St. Louis, MO 63110, USA
[20]Department of Neurological Surgery, Washington University School of Medicine, St. Louis, MO 63110, USA
[21]Department of Psychological and Brain Sciences, Washington University in St. Louis, St. Louis, MO 63130, USA
[22]Department of Psychiatry, University of Vermont, Burlington, VT 05401, USA
[23]Division of Biostatistics, University of California San Diego, La Jolla, CA 92093, USA
[24]Oxford Big Data Institute, Li Ka Shing Centre for Health Information and Discovery, Nuffield Department of Population Health, University of Oxford, Oxford, OX3 7LF, UK
[25]NUS Graduate School for Integrative Sciences and Engineering, National University of Singapore, Singapore 119077
[26]Martinos Center for Biomedical Imaging, Massachusetts General Hospital, Charlestown, MA 02129, USA
[27]Institute of Child Development, University of Minnesota Medical School, Minneapolis, MN 55455, USA
[28]Program in Occupational Therapy, Washington University School of Medicine, St. Louis, MO 63108, USA
[29]Department of Pediatrics, Washington University School of Medicine, St. Louis, MO 63110, USA
*Equal first author contribution
**Equal senior author contribution
ΩCorrespondence to:

Scott Marek, Ph.D.
Department of Psychiatry
Washington University School of Medicine
Email: smarek@wustl.edu

Brenden Tervo-Clemmens, Ph.D.
Department of Psychiatry
Massachusetts General Hospital
Harvard Medical School
Email: btervo-clemmens@mgh.harvard.edu

Damien A. Fair, PA-C, Ph.D.
Masonic Institute for the Developing Brain
University of Minnesota Medical School
Email: faird@umn.edu

Nico U.F. Dosenbach, M.D., Ph.D.
Department of Neurology
Washington University School of Medicine
Email: ndosenbach@wustl.edu

Table of Contents

Supplementary Discussion 4

Task and rest fMRI produce similar BWAS effect sizes 4

Effects of measurement reliability on BWAS 5

Supplementary Figures 7

Supplementary Fig. 1. Distributions of univariate associations between RSFC and behavioral measures. 7

Supplementary Fig. 2. Distributions of univariate BWAS effect sizes by level of analysis. 8

Supplementary Fig. 3. Inflation of univariate BWAS effects as a function of sample size. 9

Supplementary Fig. 4. Sampling variability as a function of sample size for cortical thickness. 10

Supplementary Fig. 5. Sampling variability as a function of sample size for resting-state functional connectivity (RSFC). 11

Supplementary Fig. 6. Sampling variability of associations between demographic and psychological measures (behavior-behavior). 12

Supplementary Fig. 7. Influence of current and projected future measurement reliability on BWAS effect sizes. 13

Supplementary Fig. 8. ABCD and HCP BWAS effect size histograms for NIH Toolbox subscales. 14

Supplementary Fig. 9. Statistical errors and reproducibility of univariate brain-wide associations in the UK Biobank (UKB). 15

Supplementary Fig. 10. Relationship between statistical power and sample size. 16

Supplementary Fig. 11. SVR model tuning for cortical thickness. 17

Supplementary Fig. 12. SVR model tuning for resting-state functional connectivity (RSFC). 18

Supplementary Fig. 13. Reproducibility of SVR feature weights. 19

Supplementary Fig. 14. CCA model tuning for cortical thickness. 20

Supplementary Fig. 15. CCA Model tuning for resting-state functional connectivity (RSFC). 21

Supplementary Fig. 16. Multivariate BWAS effect size inflation as a function of sample size. 22

Supplementary Fig. 17. Group average RSFC requires substantially smaller sample sizes to achieve excellent reproducibility. 23

Supplementary Tables 24

Supplementary Table 1. ABCD behavioral measures. 24

Supplementary Table 2. Probability of out-of-sample replication as a function of statistical threshold (multivariate associations). 26

Supplementary Table 3. Human Connectome Project (HCP) task contrasts. 27

Supplementary Table 4. Human Connectome Project (HCP) psychological measures. 28

Supplementary Discussion

Task and rest fMRI produce similar BWAS effect sizes

In addition to structural brain measures and resting state functional connectivity (RSFC), functional MRI (fMRI) task activations have also frequently been correlated with behavioral measures[1]. Individual differences in task activations are thought to be a combination of task-specific processes (e.g., working memory task accuracy) and more general behavioral phenotypes (e.g., cognitive ability). Using HCP data (n = 844; 56 participants excluded from the n = 900 due to missing data), we assessed the effect sizes for associations between individual differences in task activation (fMRI) and behavioral phenotypes[2] (see Supplementary Tables 3, 4 for the HCP task contrasts and phenotypes, respectively)[1,3].

We observed univariate associations between task activations and related out of scanner cognitive ability, on par with those previously reported[1]. Specifically, the correlation between working memory (WM; 2back vs 0back) task activation in the dorsal attention network and cognitive ability (NIH Toolbox) was r = 0.34 (Extended Data Fig. 3a). However, in-scanner working memory performance (WM) and the broader behavioral phenotype (cognitive ability) were even more strongly correlated at r = 0.54 (Extended Data Fig. 3b). Also, task performance is robustly related to task-induced activation[4]. Thus, specific behavioral performance differences (WM), inflate the association between task activation and the cognitive ability phenotype. Task performance should therefore be treated as a covariate to deconfound the association between task activation and out-of-scanner behavioral phenotypes. In this example, WM accuracy must be regressed out of the general behavioral phenotype (cognitive ability). After controlling for WM accuracy, the association between WM task fMRI activations and cognitive ability was considerably reduced (r = 0.14; Extended Data Fig. 3c), but still slightly stronger than the RSFC association (r = 0.11).

After controlling for accuracy during each task, the top 1% largest task fMRI/phenotype associations across all tasks and phenotypes reached $|r| > 0.10$ (vs. HCP RSFC top 1% largest $|r|$ > 0.11), revealing equivalent BWAS effect sizes when using fMRI task activations as a functional brain measure instead of RSFC (Extended Data Fig. 3d). The apparent strengthening of brain-behavior associations with fMRI task activations (Extended Data Fig. 3a) was specific to those psychological phenotypes (e.g., cognitive ability) that overlapped with the task of interest (e.g., working memory).

It has long been appreciated that fMRI task activations index specific task performance[4]. Cognitive ability includes a working memory component. Thus predicting cognitive ability from working memory task activation, is equivalent to including memory behavioral scores in models predicting cognitive ability from structural/RSFC MRI data. Accounting for task performance effects resulted in near identical brain-behavior effect size distributions for task activations and

RSFC. Therefore, relating fMRI task activations to cognitive phenotypes, across participants, after the removal of performance effects, could be considered a form of BWAS. However, similar analyses that do not remove task specific performance effects conflate classic task fMRI predictions and out-of-scanner performance and BWAS. These approaches will thus have a predictive bias towards phenotypes that overlap with or are indicated by the specific task process (e.g., working memory tasks with cognitive ability).

Effects of measurement reliability on BWAS

Measurement reliability (precision) can affect observed BWAS effects. High measurement error of brain structure/function and/or of cognitive/mental health phenotypes may attenuate observed correlations. Classic psychometrics[5] formalizes the relationship between reliability, observed correlation, and theoretical/latent correlation in the following equation:

$$r_{x'}r_{y'} = \frac{r_{xy}}{\sqrt{r_{xx}r_{yy}}}$$

where $r_{x'}r_{y'}$ is the disattenuated or theoretical correlation between variable x and variable y, corrected for measurement error, r_{xy} is the observed correlation, and r_{xx} and r_{yy} are the reliabilities of variables x and y, respectively.

Using this formula, we estimated how potential future improvements in measurement reliability could allow for larger BWAS effect sizes. We visualized the relationship between measurement reliability and univariate BWAS effect sizes with a measurement reliability lower bound of $r = 0.5$, for median BWAS effect sizes ($r = 0.01$; Supplementary Fig. 7, left panel) and the top 1% largest ($r = 0.06$; Supplementary Fig. 7, right panel). The measurement reliability lower bound of $r = 0.5$ was based on our own split-half reliability analyses of ABCD RSFC data (see Extended Data Fig. 4) and emerging consensus on the reliabilities of brain structure[6]/function[7] and the included cognitive[8] and mental health[9] phenotypes. For ease of interpretation, we present plots assuming brain and behavioral measures have the same reliability (Supplementary Fig. 7). These results suggest that small univariate BWAS effects ($r = 0.01$) would not benefit from improved measurement reliability. In contrast, larger effects ($r = 0.06$; top 1% largest univariate) could theoretically benefit from potential future improvements in measurement reliability. For example, a BWAS of $r = 0.06$ obtained when both the brain and behavioral metric were measured with reliability of 0.5, could grow to $r = 0.12$, if both brain and behavioral metrics were measured with perfect reliability. If current measurement reliability is already better than 0.5, then the expected benefits from improved measurement are diminished. Hence, we note that the NIH Toolbox and CBCL total problem scores highlighted in the current analyses have been shown to have much higher reliability (~0.9) in validation samples [8,9], which when paired with the ABCD Study RSFC reliability (~0.5, Extended Data Fig. 4) would suggest a BWAS of $r = 0.06$ could grow to a maximum of 0.09 with perfect measurement of behavior and RSFC. Projected maximum BWAS effect sizes will likely never be reached due to

fundamental biological limits on the strength of the true association and/or limits imposed by MR physics/behavioral phenotyping measurement precision. Potentially greater effect sizes would still be within the range of the maximum observed BWAS effect size ($r = 0.14$) and thus require large sample sizes.

The theoretical maximum BWAS effect sizes shown in Supplementary Fig. 7 are specific to univariate analyses. Unfortunately, an analogous formula describing the relationship between multivariate BWAS and measurement error does not exist. Given the strong correlation between univariate and multivariate BWAS effects ($r = 0.79$; Fig. 4f), improvements in measurement reliability could also slightly increase some of the replicable multivariate BWAS effect sizes[10].

Supplementary Figures

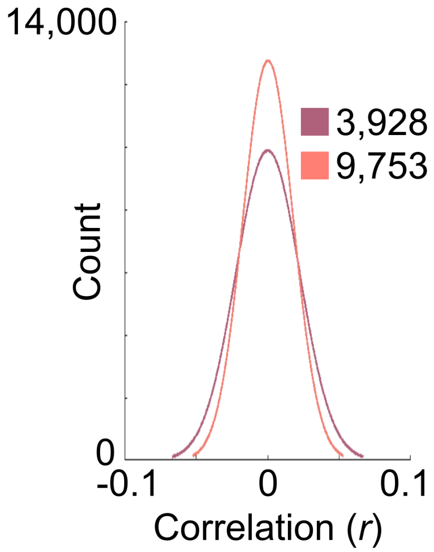

Supplementary Fig. 1. Distributions of univariate associations between RSFC and behavioral measures.

Comparison between strictly denoised ABCD sample (*n* = 3,928; >8 min. RSFC data; edges) and the minimally-denoised ABCD sample (*n* = 9,753; no RSFC data minimum; edges), across all associations.

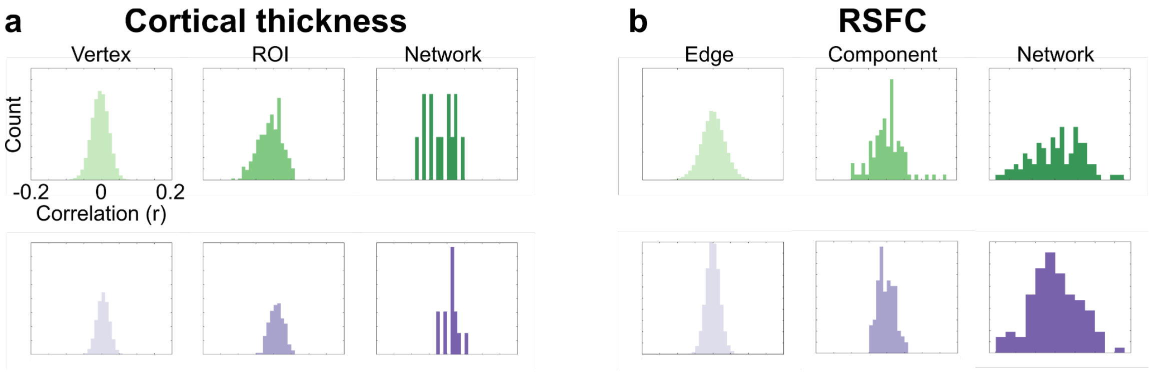

Supplementary Fig. 2. Distributions of univariate BWAS effect sizes by level of analysis. Histograms of all **(a)** cortical thickness associations with cognitive ability (green) and psychopathology (purple) and **(b)** RSFC associations with cognitive ability (green) and psychopathology (purple). Phenotypic correlations with brain measures were generated across multiple levels of scale (cortical thickness: vertices, ROIs, networks; RSFC: ROI-ROI pairs [edges], principal components, networks), which are represented in varying shades of green and purple to match Fig. 1a,b. Each paneled histogram contains the same x-axis as the upper leftmost panel. All data shown are from the ABCD Study (n = 3,928).

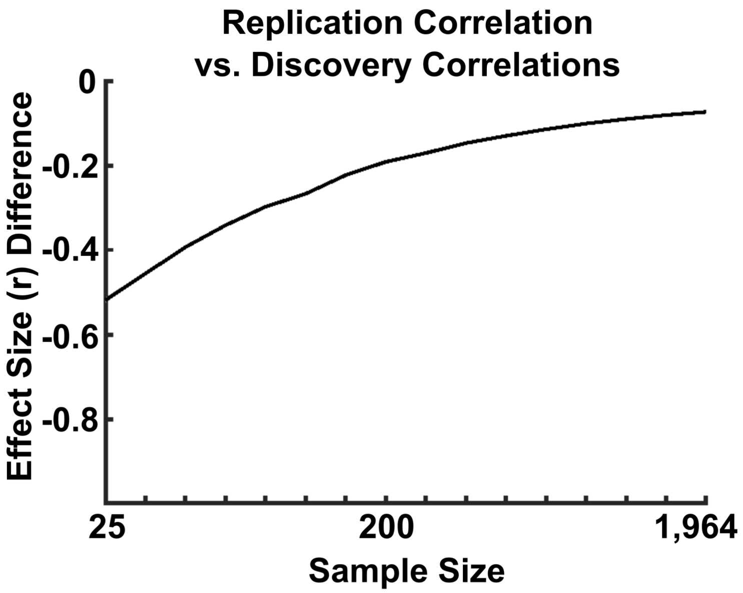

Supplementary Fig. 3. Inflation of univariate BWAS effects as a function of sample size.

For the top 1% largest univariate BWAS effects, the difference between the in-sample effect and the corresponding out-of-sample effect (replication set *r* - discovery set *r*) are shown. The top 1% largest effect sizes were determined for each demographic/psychological phenotypes (cf. Supplementary Table 1) across 100 resampled in-sample datasets and compared to an equivalently sized out-of-sample set. At the largest sample size (n = 1,964), out-of-sample correlations were 78% smaller (r = 0.07) in the replication dataset, than in the discovery set (in-sample).

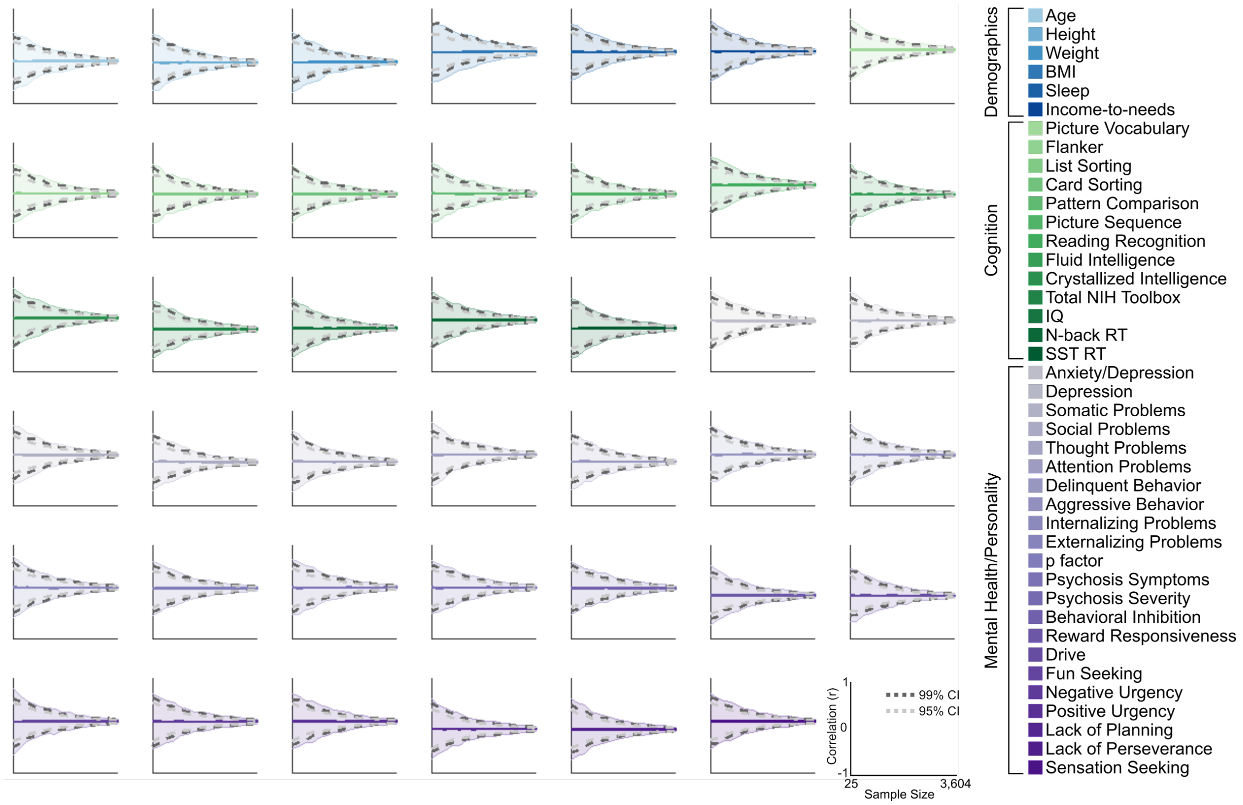

Supplementary Fig. 4. Sampling variability as a function of sample size for cortical thickness.

Sampling variability of the correlation between cortical thickness and each demographic, cognitive and mental health measure (*n* = 41). For each brain-wide association, 16,000 total resampled subsamples (1,000 subsamples for each sample size) were generated. Across sample sizes, sampling variability of the largest brain-wide association is depicted as the range of observable correlations (shaded area), as well as the 99% confidence interval (dark gray) and 95% confidence interval (light gray). For each sampling bin, the dark colored line represents the mean brain-wide association across the 1,000 resamples. The ordering of subgraphs follows the ordering of measures in the legend. All data shown are from the ABCD Study (*n* = 3,604, cortical thickness only).

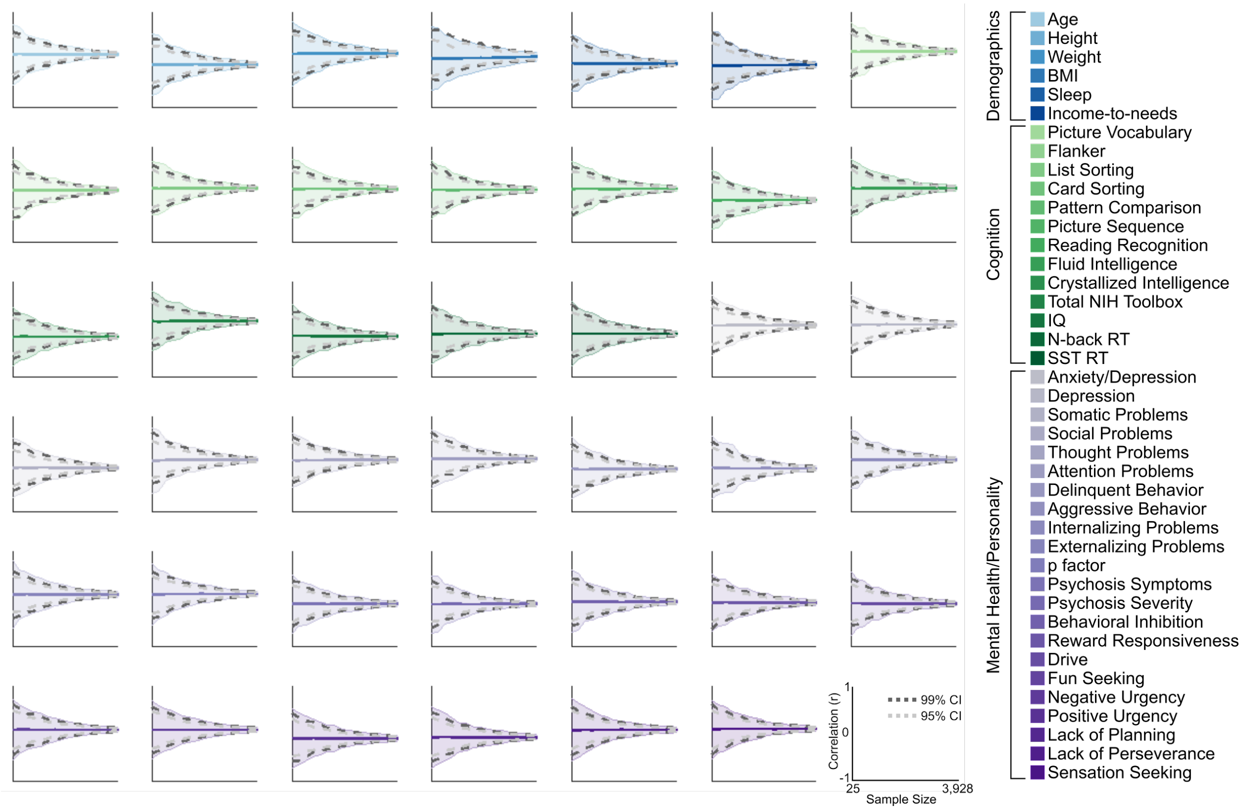

Supplementary Fig. 5. Sampling variability as a function of sample size for resting-state functional connectivity (RSFC).

Sampling variability of the correlation between RSFC and each demographic, cognitive and mental health measure (n = 41). For each brain-wide association, 16,000 resampled studies (1,000 subsamples for each sample size) were generated. Across sample sizes, sampling variability of the largest brain-wide association is depicted as the range of observable correlations (shaded area), as well as the 99% confidence interval (dark gray) and 95% confidence interval (light gray). For each sampling bin, the dark colored line represents the mean brain-wide association across the 1,000 resamples. The ordering of subgraphs follows the ordering of measures in the legend. All data shown are from the ABCD Study (n = 3,928).

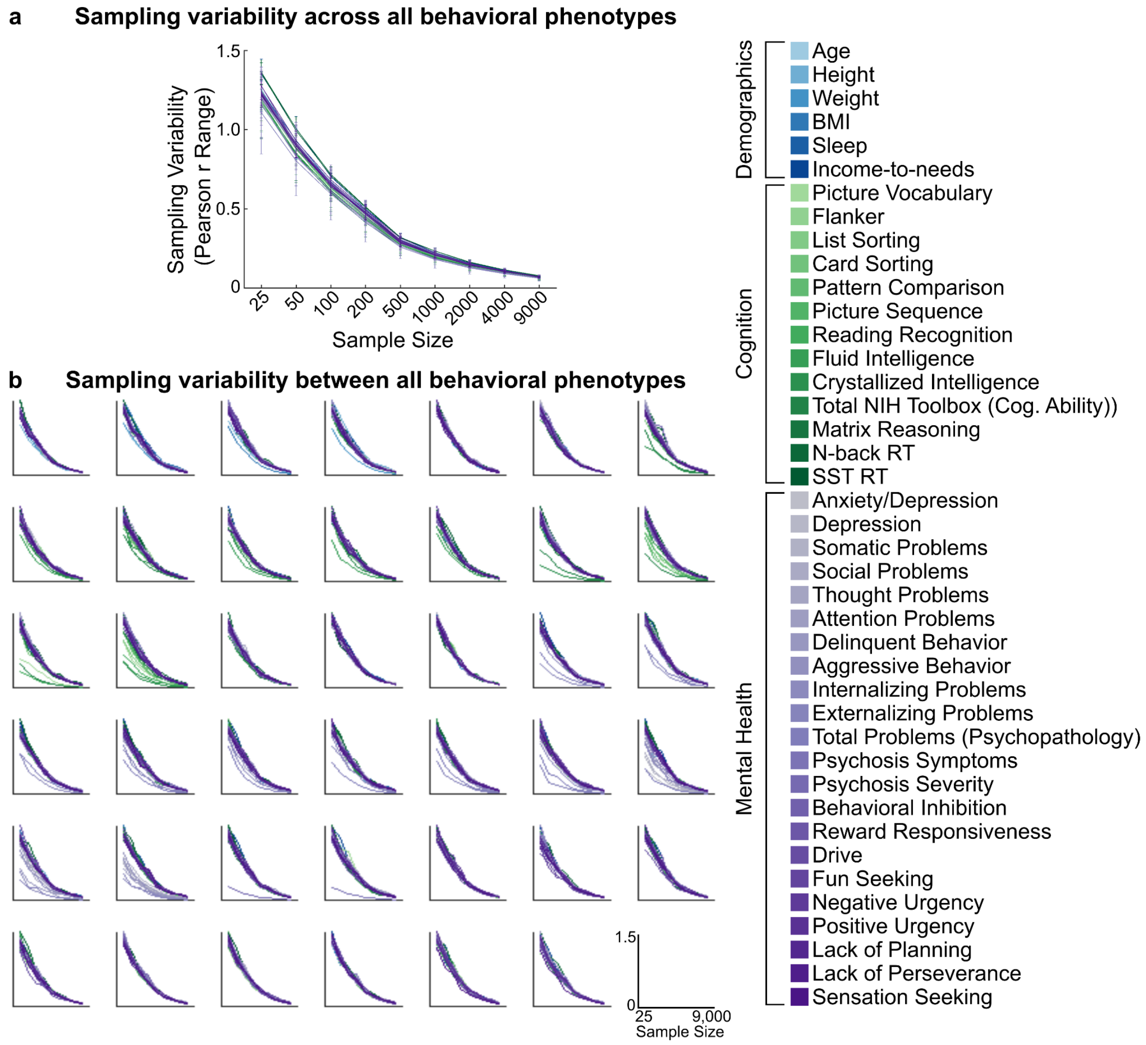

Supplementary Fig. 6. Sampling variability of associations between demographic and psychological measures (behavior-behavior).

(a) Sampling variability (range of observable correlations) as a function of sample size (sampling bins: n = 25, 50, 100, 200, 500, 1,000, 2,000, 4,000, 9,000). Each line represents the average sampling variability for the correlation between the given measure (demographic, cognition, mental health; see color code) and all other behavioral measures. Error bars denote one standard deviation across all behavioral measures. Note that sampling variability ranges from 0-2 for bivariate linear correlations, given observables correlations range from -1 to 1. **(b)** Each subgraph depicts sampling variability as a function of sample size for a given demographic or psychological measure (n = 41) to every other demographic/psychological measure. The ordering of subgraphs follows the ordering of measures in the legend (e.g., the most top-left panel shows sampling variability between Age and every other demographic and psychological measure). Each curve is colored corresponding to the measures in the figure legend. All data shown are from the ABCD Study (n = 9,000).

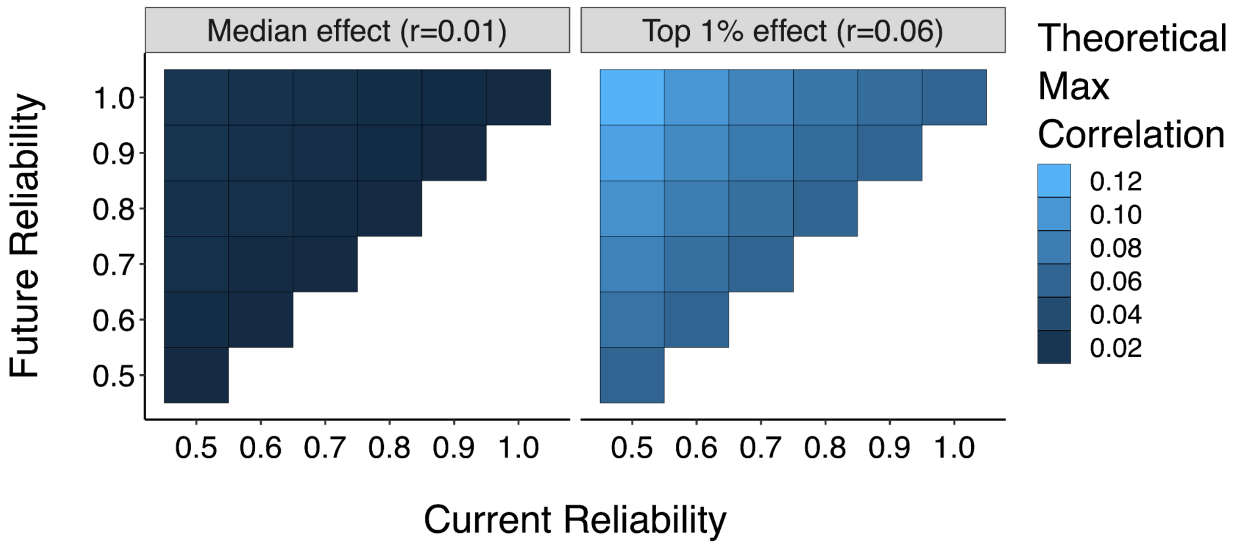

Supplementary Fig. 7. Influence of current and projected future measurement reliability on BWAS effect sizes.

Y-axis displays simulated assumed future reliability of both neuroimaging and behavioral measure (see Supplementary Discussion). X-axis displays assumed current reliability (neuroimaging and behavioral measures) for $r = 0.01$ (median BWAS effect size; left) and $r = 0.06$ (top 1% BWAS effect size; right). Color denotes the theoretical maximum correlation based on the disattenuation formula (see Supplementary Discussion). For ease of interpretation, behavior and imaging reliability are assumed to be the same in these plots. Please see Supplemental Discussion for discussion on current estimates of behavioral and neuroimaging reliability.

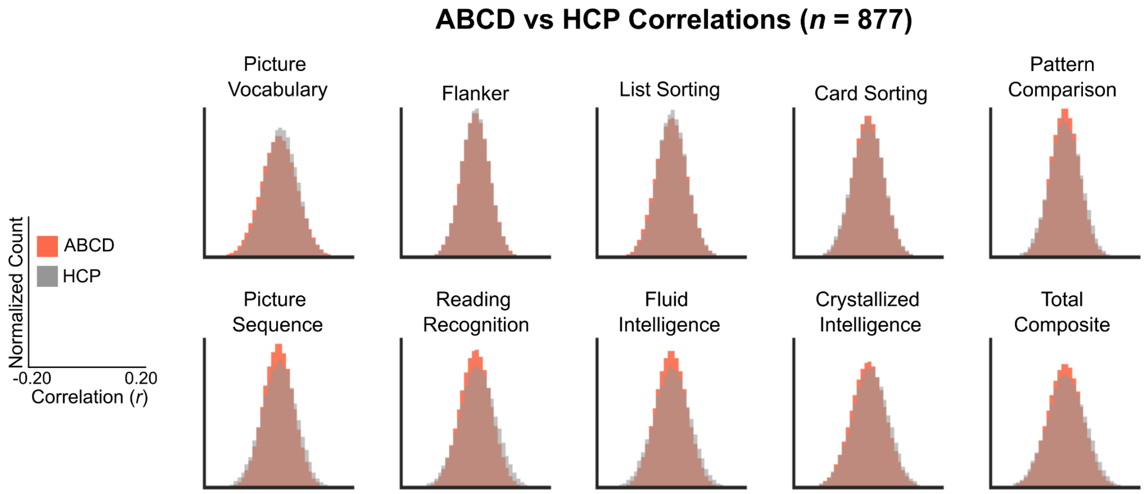

Supplementary Fig. 8. ABCD and HCP BWAS effect size histograms for NIH Toolbox subscales.

Effect sizes observed in subsamples of ABCD (n = 877) replicate full sample brain-phenotype correlations from HCP (n = 877). ABCD data were subsampled to match the sample size of HCP. Data were subsampled 100 times. For each NIH Toolbox subscale the correlation between every ROI pair and phenotype was generated for HCP data and each of the 100 resamples of ABCD data. Each histogram (ABCD: red; HCP: gray) was generated from these ROI-ROI pair brain-phenotype associations.

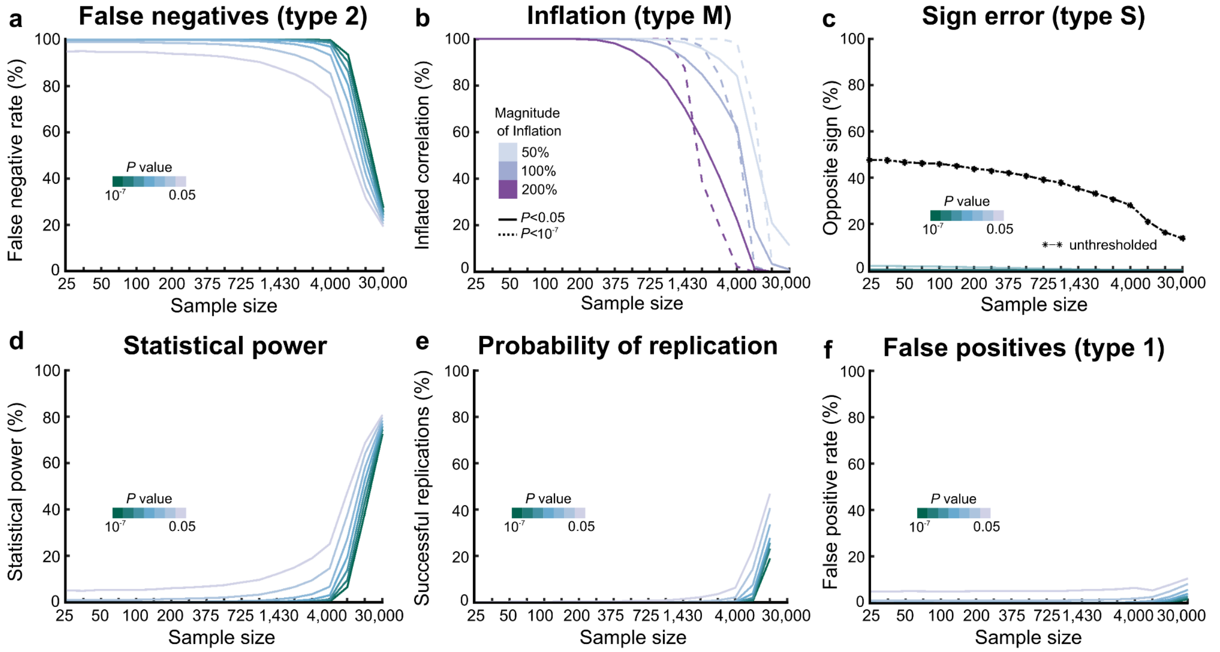

Supplementary Fig. 9. Statistical errors and reproducibility of univariate brain-wide associations in the UK Biobank (UKB).

(a) False negative rates (y-axis; relative to full sample: see Methods) for correlations (*r*; bivariate linear) between psychological phenotypes and brain features (cortical thickness: vertex-wise; resting-state functional connectivity [RSFC]: edge-wise), as a function of sample size (x-axis) and *P* value (color scale; *P* values [all two-tailed]: <0.05, $<10^{-2}$,$<10^{-3}$, $<10^{-4}$,$<10^{-5}$, $<10^{-6}$,$<10^{-7}$; *P* value thresholding was identical in full sample and subsamples). These P values were also used in panels **c-f** **(b)** Magnitude error rates (y-axis) for three levels of effect size inflation (50%, 100%, 200%) as a function of sample size (x-axis) and statistical threshold ($P < 0.05$ and $P < 10^{-7}$, same in full sample and subsample). Line color represents the inflation level (solid line: $P < 0.05$; dashed line: Bonferroni correction [$P < 10^{-7}$]; starred line: no statistical thresholding). **(c)** Sign error rates (y-axis) representing the percentage of subsamples with the opposite sign of the full sample, as a function of sample size (x-axis) and *P* value (color scale). Starred line represents sign error rates with no statistical thresholding. **(d)** Statistical power of subsamples relative to full sample (y-axis; same sign, both significant) as a function of sample size (x-axis) and *P* value (color scale). **(e)** Probability (%; y-axis) of replicating (same sign, both significant) a univariate brain-phenotype association out-of-sample across *P* values (color scale; note: data ends at $n \sim 2{,}000$ as the replication sample is half of the full sample). Replication rates comport with the square of power. **(f)** False positive rates (y-axis) of subsamples relative to full sample, as a function of sample size (x-axis) and *P* value (color scale).

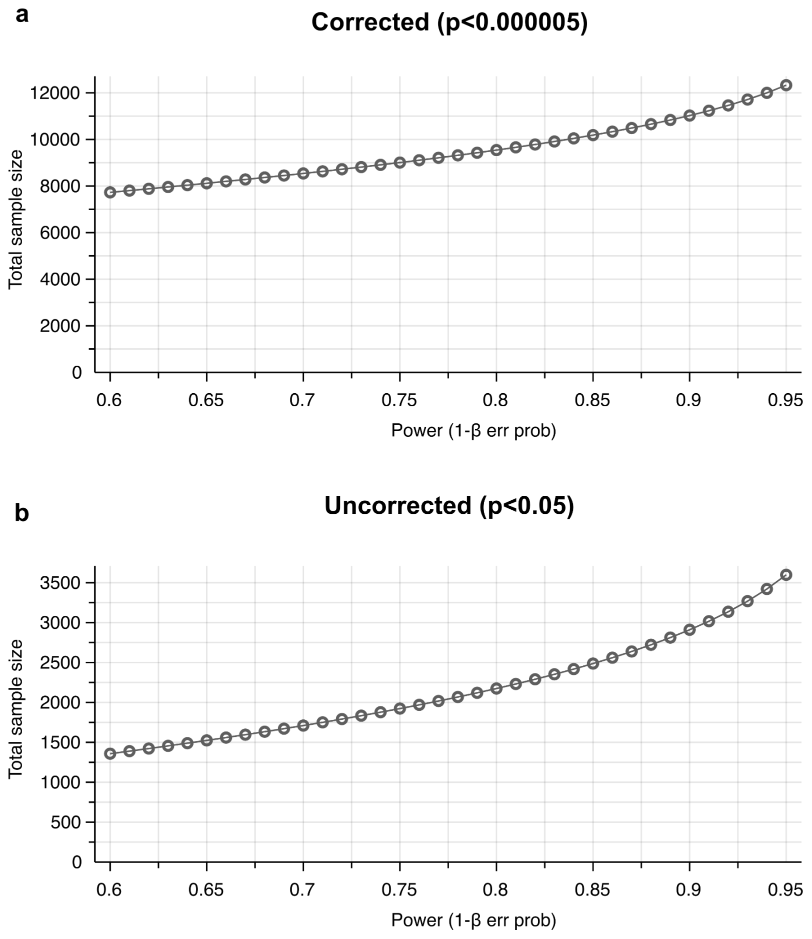

Supplementary Fig. 10. Relationship between statistical power and sample size.

Analytic requisite sample sizes to detect 99th percentile (largest 1%; $r = 0.06$) univariate brain-wide associations at **(a)** $P < 10^{-7}$ (i.e., $P < 0.05$, Bonferroni-corrected, two-sided) and **(b)** $P <$ 0.05 (uncorrected, two-sided).

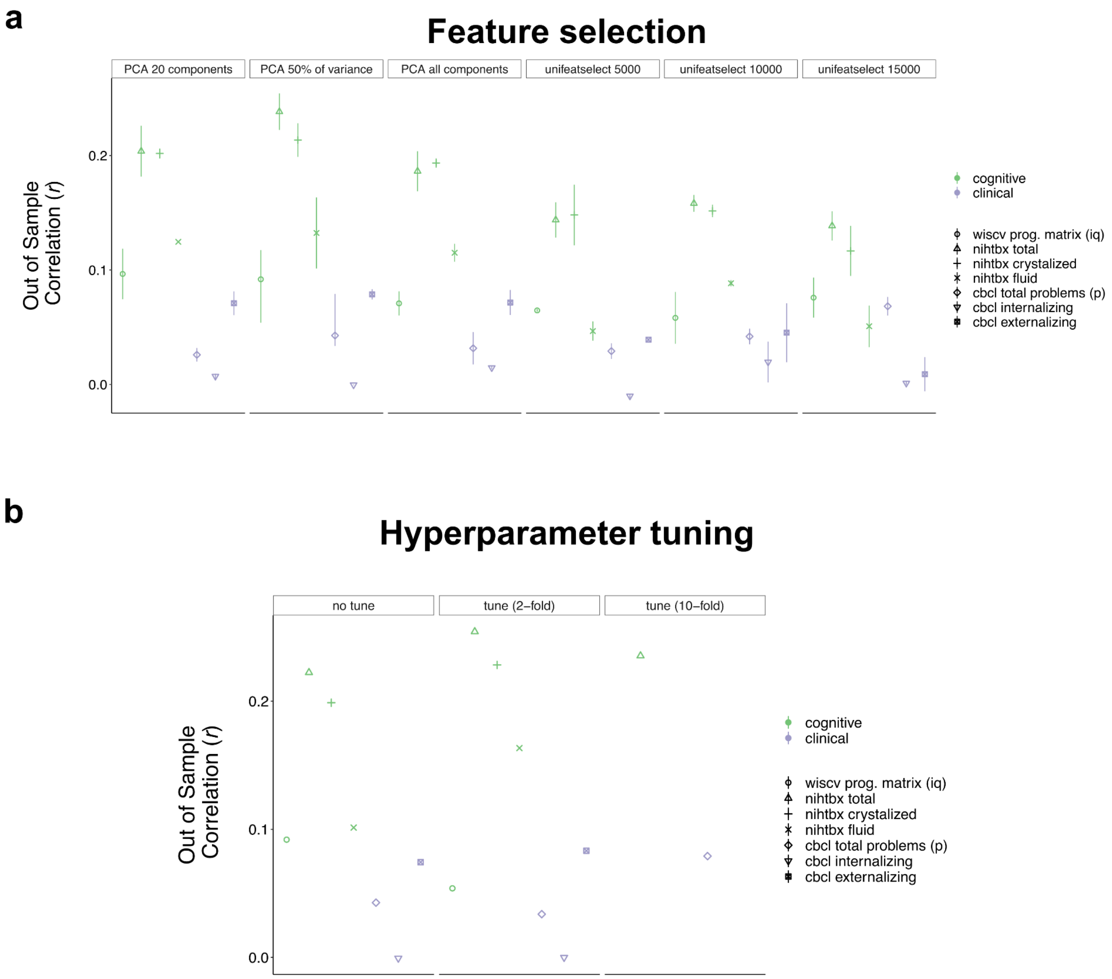

Supplementary Fig. 11. SVR model tuning for cortical thickness.

Out-of-sample associations (r_{pred}) for cortical thickness associations with phenotypes trained on the full ABCD discovery set (n = 1,814) and tested on the full replication set (n = 1,790) using support vector regression (SVR) across **(a)** feature selection procedure (principal components left, univariate feature ranking right) and **(b)** hyperparameter tuning. Note, error bars/point range in **(a)** shows min/max across tuned and non-tuned models from **(b)**; **(b)** displays tuned and non-tuned models using PCA with 50% of the variance retained.

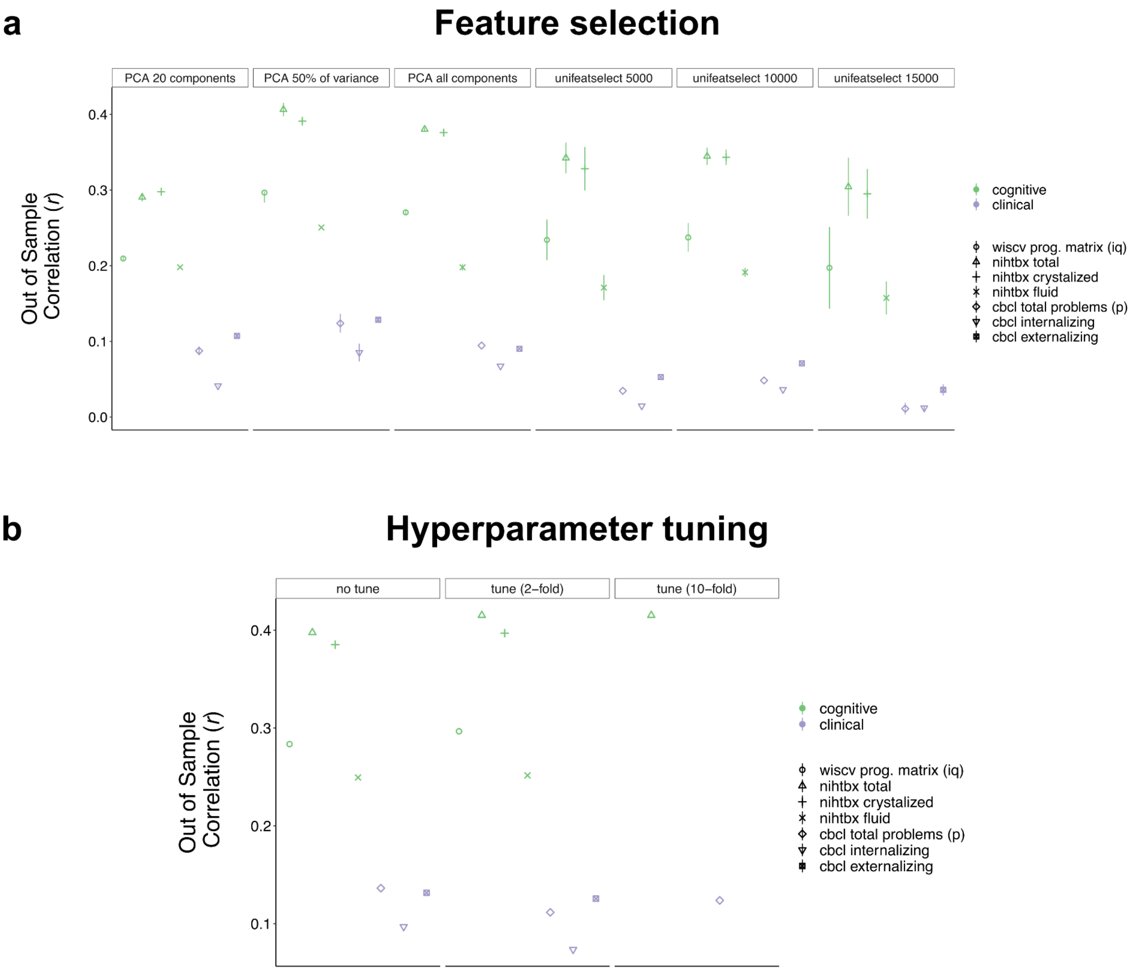

Supplementary Fig. 12. SVR model tuning for resting-state functional connectivity (RSFC). Out-of-sample associations (r_{pred}) for RSFC associations with phenotypes trained on the full ABCD discovery set (n = 1,964) and tested on the full replication set (n = 1,964) using support vector regression across **(a)** feature selection procedure (principal components left, univariate feature ranking right) and **(b)** hyperparameter tuning. Note, error bars/point range in **(a)** shows min/max across tuned and non-tuned models from **(b)**; **(b)** displays tuned and non-tuned models using PCA with 50% of the variance retained.

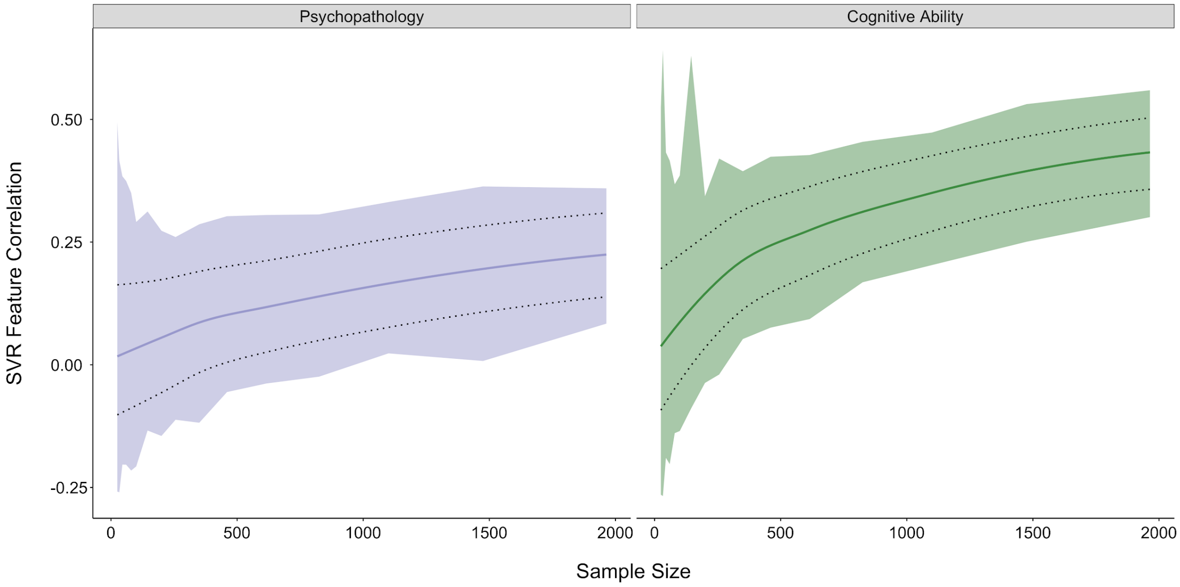

Supplementary Fig. 13. Reproducibility of SVR feature weights.

Correlations across RSFC feature weights from 100 bootstrapped subsamples (with replacement) as a function of sample size for psychopathology (left; purple) and cognitive ability (right; green), in ABCD data. The colored area displays the range of values (psychopathology purple; cognitive ability green); the dotted lines show the 95% confidence interval. Solid lines represent the mean across the 100 bootstrapped subsamples.

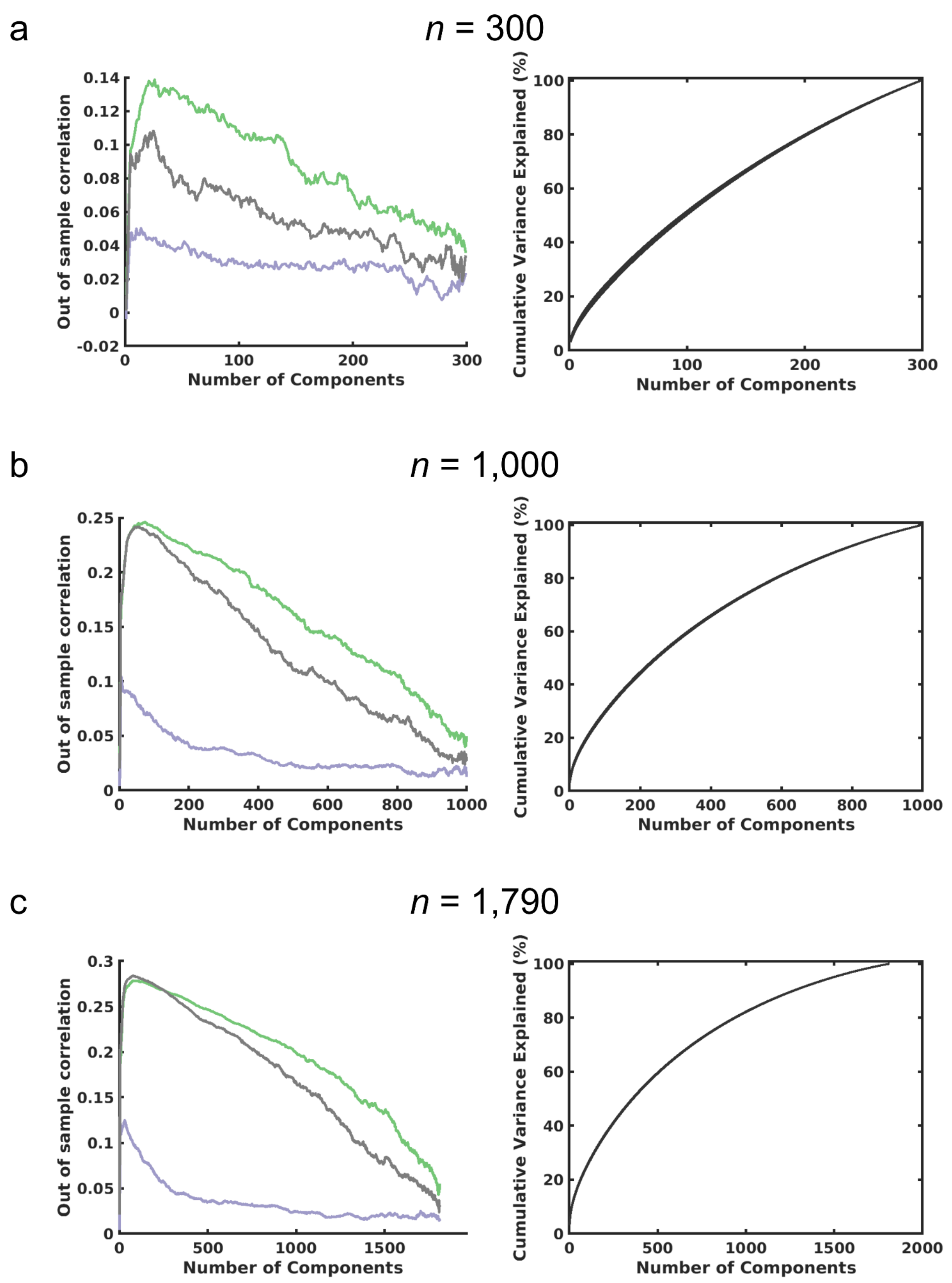

Supplementary Fig. 14. CCA model tuning for cortical thickness.

Out-of-sample associations (r_{CV1}) as a function of the number of principal components included in cortical thickness CCA models for cognition (green), psychopathology (purple), and when combining cognition and psychopathology (grey). Across three distinct sample sizes (**a**: *n* = 300, **b**: *n* = 1,000, and **c**: *n* = 1,790; 100 iterations of each), ~20% of the cumulative principal component variance maximized the out-of-sample correlation. Note x- and y-axes are scaled to fit data.

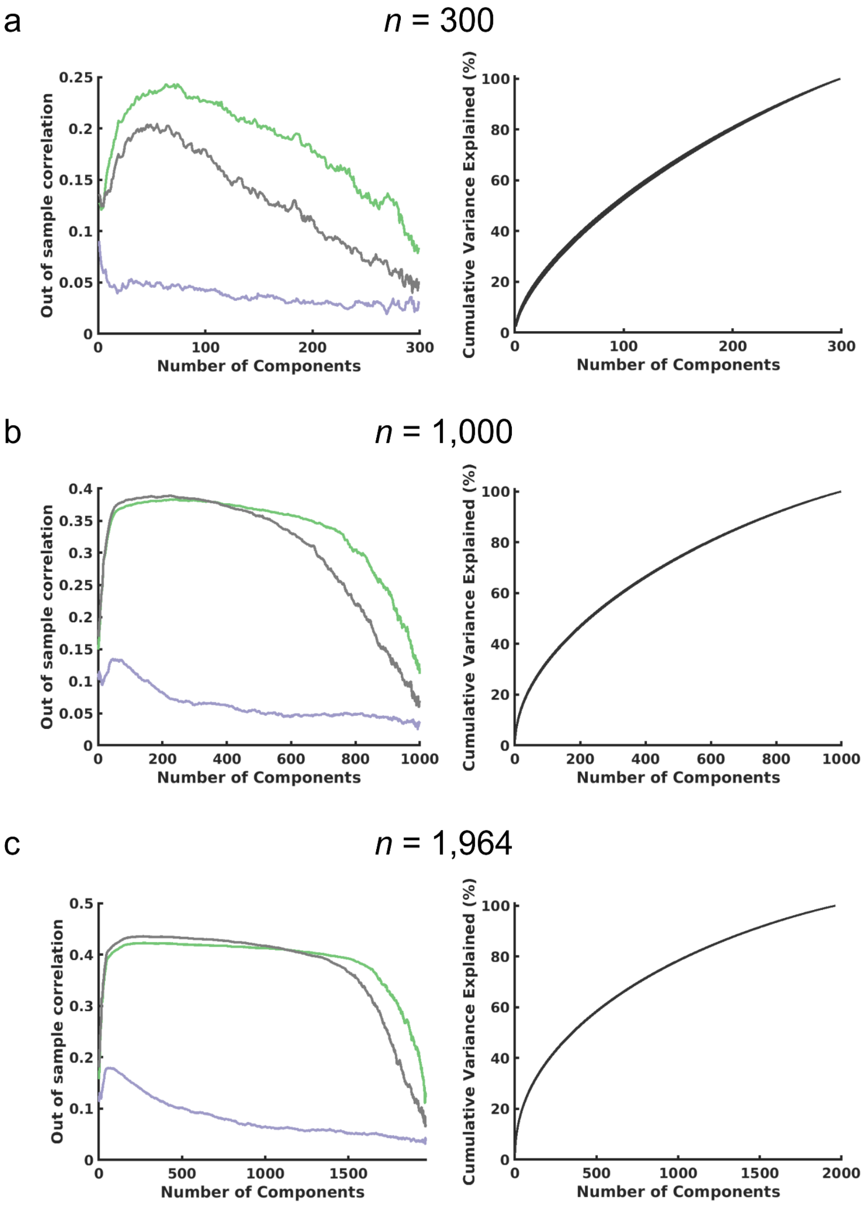

Supplementary Fig. 15. CCA Model tuning for resting-state functional connectivity (RSFC).

Out-of-sample associations (r_{CV1}) as a function of the number of principal components included in RSFC CCA models for cognition (green), psychopathology (purple), and when combining cognition and psychopathology (grey). Similar to cortical thickness, across three distinct sample sizes (**a**: $n = 300$, **b**: $n = 1{,}000$, and **c**: $n = 1{,}964$; 100 iterations of each), ~20% of the cumulative principal component variance maximized the out-of-sample correlation. Note x- and y-axes are scaled to fit data.

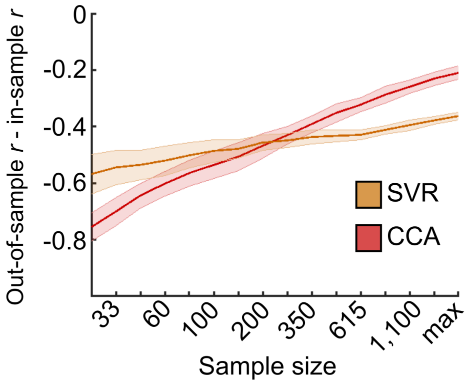

Supplementary Fig. 16. Multivariate BWAS effect size inflation as a function of sample size.

Effect size inflation (out-of-sample *[*r_{pred}, r_{CV1}*]* - in-sample *[*r_{pred}, r_{CV1}*]*) for SVR (orange) and CCA (red) in ABCD, as a function of sample size. Solid line represents the mean and shading represents one standard deviation around the mean. Maximum sample size for cortical thickness was n = 1,790 and for RSFC was n = 1,964.

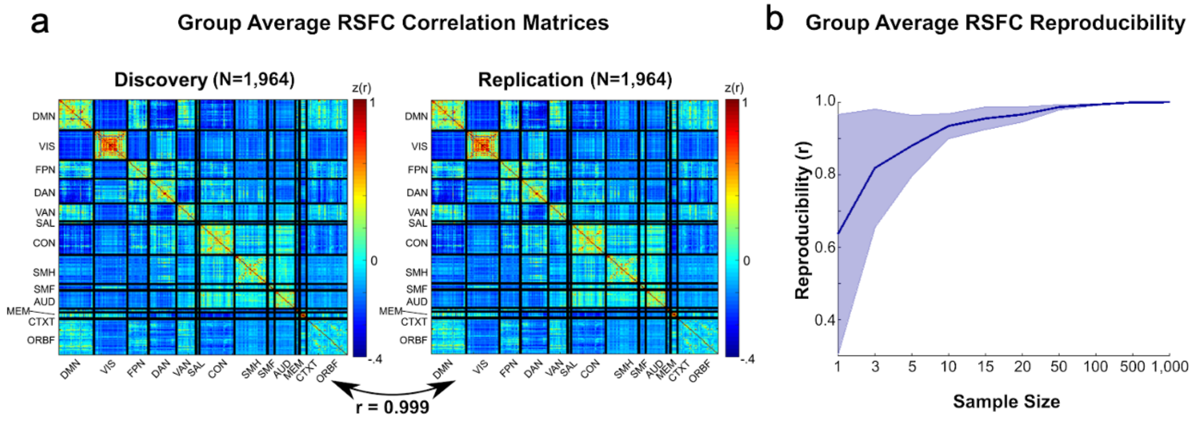

Supplementary Fig. 17. Group average RSFC requires substantially smaller sample sizes to achieve excellent reproducibility.

(a) Group average RSFC matrices from the ABCD discovery (left) and replication data sets (right). Both datasets contain n = 1,964. The spatial correlation between each full group average is $r > 0.999$. **(b)** Reproducibility of group-average discovery RSFC correlation matrix with the Replication dataset as a function of sample size. A group average matrix was generated from the discovery dataset. The Replication dataset was subsampled (1,000 x at each sample size [n = 1, 3, 5, 10, 15, 20, 50, 100, 500, 1,000]), averaged, and correlated to the group average discovery set matrix. Dark blue line represents the mean across the 1,000 resamples. Shaded error bar represents the 99% confidence interval.

Supplementary Tables

Supplementary Table 1. ABCD behavioral measures.

Original ABCD variable names with the corresponding descriptive labels used in the manuscript. More details on the demographic and psychological measures can be found in the ABCD data dictionary.

**Description**	**ABCD field**
Age	interview_age
Height	anthro_height_calc
Weight	anthro_weight_calc
BMI	703*weight/(height^2)
Sleep	prescan_state_sleepy_1
Income-to-needs	Demo_comb_income_v2b/demo_roster_p
Vocabulary	nihtbx_picvocab_agecorrected
Attention	nihtbx_flanker_agecorrected
Working memory	nihtbx_list_agecorrected
Executive function	nihtbx_cardsort_agecorrected
Processing speed	nihtbx_pattern_agecorrected
Episodic memory	nihtbx_picture_agecorrected
Reading	nihtbx_reading_agecorrected
Fluid intelligence	nihtbx_fluidcomp_agecorrected
Crystallized intelligence	nihtbx_cryst_agecorrected
Cognitive ability	nihtbx_totalcomp_agecorrected
Matrix reasoning	pea_wiscv_tss
N-back RT	tfmri_nback_all_beh_correct.total_mean_rt

SST RT	tfmri_sst_all_beh_correct.go.mean.rt
Anxious depressed	cbcl_scr_syn_anxdep_t
Withdrawn depressed	cbcl_scr_syn_withdep_t
Somatic complaints	cbcl_scr_syn_somatic_t
Social problems	cbcl_scr_syn_social_t
Thought problems	cbcl_scr_syn_thought_t
Attention problems	cbcl_scr_syn_attention_t
Rule-breaking behavior	cbcl_scr_syn_rulebreak_t
Aggressive behavior	cbcl_scr_syn_aggressive_t
Internalizing	cbcl_scr_syn_internal_t
Externalizing	cbcl_scr_syn_external_t
Psychopathology	cbcl_scr_syn_totalprob_t
Psychosis symptoms	pps_y_ss_number
Psychosis severity	pps_y_ss_severity_score
Behavioral inhibition	bis_y_ss_bis_sum
Reward responsiveness	bis_y_ss_bas_rr
Drive	bis_y_ss_bas_drive
Fun seeking	bis_y_ss_bas_fs
Negative urgency	upps_y_ss_negative_urgency
Positive urgency	upps_y_ss_positive_urgency
Lack of planning	upps_y_ss_lack_of_planning
Lack of perseverance	upps_y_lack_of_perseverance
Sensation seeking	upps_y_ss_sensation_seeking

Supplementary Table 2. Probability of out-of-sample replication as a function of statistical threshold (multivariate associations).

For each imaging modality (cortical thickness [CT], RSFC), psychological phenotype in the main analysis (cognitive ability [Tlbx], general psychopathology [CBCL]), and multivariate approach (SVR, CCA), we examined the top 5% strongest in-sample associations and quantified the percentage of models that passed the 99% confidence interval of the null model, as a function of sample size (*n*). Maximum sample size for cortical thickness was *n* = 1,790 and for RSFC was *n* = 1,964.

n	**CCA/RSFC/ Tlbx**	**CCA/RSFC/ CBCL**	**CCA/CT/ Tlbx**	**CCA/CT/ CBCL**	**SVR/CT/ Tlbx**	**SVR/CT/ CBCL**	**SVR/RSFC /Tlbx**	**SVR/RSFC /CBCL**
**25**	0	0	0	0	0	0	40	0
**33**	20	0	0	0	0	0	60	0
**45**	40	0	0	0	20	0	60	0
**60**	40	0	0	0	40	0	100	0
**80**	40	0	0	0	60	0	100	0
**100**	40	0	20	0	80	0	100	0
**145**	60	0	20	0	100	0	100	0
**200**	60	0	20	0	100	0	100	0
**256**	60	0	40	0	100	0	100	0
**350**	60	0	80	0	100	0	100	0
**460**	80	20	100	0	100	0	100	0
**615**	100	20	100	0	100	0	100	0
**825**	100	20	100	0	100	0	100	20
**1100**	100	20	100	0	100	0	100	20
**1475**	100	40	100	20	100	0	100	20
**Max**	100	60	100	20	100	0	100	40

Supplementary Table 3. Human Connectome Project (HCP) task contrasts.

Task	Contrasts
Emotion	Faces, Shapes, Faces-Shapes, neg_Faces, neg_Shapes, Shapes-Faces
Gambling	Punish, Reward, Punish-Reward, neg_Punish, neg_Reward, Reward-Punish
Language	Math, Story, Math-Story, Story-Math, neg_Math, neg_Story,
Motor	Cue, LF, LH, RF, RH, T, Avg, Cue-Avg, LF-Avg, LH-Avg, RF-Avg, RH-Avg, T-Avg, neg_Cue, neg_LF, neg_LH, neg_RF, neg_RH, neg_T, neg_Avg, Avg-Cue, Avg-LF, Avg-LH, Avg-RF, Avg-RH, Avg-T
Relational	Match, Rel, Match-Rel, Rel-Match, neg_Match, neg_Rel
Social	Random, Tom, Random-Tom, neg_Random, neg_Tom, Tom-Random
Working Memory	2BK_Body, 2BK_Face, 2BK_Place, 2BK_Tool, 0BK_Body, 0BK_Face, 0BK_Place, 0BK_Tool, 2BK, 0BK, 2BK-0BK, neg_2BK, neg_0BK, 0BK-2BK, Body, Face, Place, Tool, Body-Avg, Face-Avg, Place-Avg, Tool-Avg, neg_Body, neg_Face, neg_Place, neg_Tool, Avg-Body, Avg-Face, Avg-Place, Avg-Tool

Supplementary Table 4. Human Connectome Project (HCP) psychological measures.

Domain	Description	HCP field
Cognition	Vocabulary	PicVocab_AgeAdj
Cognition	Attention	Flanker_AgeAdj
Cognition	Working memory	ListSort_AgeAdj
Cognition	Executive function	CardSort_AgeAdj
Cognition	Processing speed	ProcSpeed_AgeAdj
Cognition	Episodic memory	PicSeq_AgeAdj
Cognition	Reading	ReadEng_AgeAdj
Cognition	Fluid intelligence	CogFluidComp_AgeAdj
Cognition	Crystallized intelligence	CogCrystalComp_AgeAdj
Cognition	Cognitive ability	CogTotalComp_AgeAdj
Emotion	Sadness	Sadness_Unadj
Emotion	Fear - Affect	FearAffect_Unadj
Emotion	Fear - Somatic Arousal	FearSomat_Unadj
Emotion	Anger - Affect	AngAffect_Unadj
Emotion	Anger - Physical Aggression	AngAggr_Unadj
Emotion	Anger - Hostility	AngHostil_Unadj
Emotion	Life Satisfaction	LifeSatif_Unadj
Emotion	Meaning and Purpose	MeanPurp_Unadj
Emotion	Positive Affect	PosAffect_Unadj
Emotion	Emotional Support	EmotSupp_Unadj
Emotion	Friendship	Friendship_Unadj
Emotion	Perceived Hostility	PercHostil_Unadj
Emotion	Perceived Rejection	PercReject_Unadj
Emotion	Perceived Stress	PercStress_Unadj

Emotion	Self Efficacy	SelfEff_Unadj
Motor	Dexterity	Dexterity_AgeAdj
Motor	Endurance	Endurance_AgeAdj
Motor	Grip Strength	Strength_AgeAdj
Motor	Gait Speed	GaitSpeed_Comp
Sensory	Words in Noise	Noise_Comp
Sensory	Smell	Odor_AgeAdj
Sensory	Pain Interference	PainInterf_Tscore
Sensory	Taste	Taste_AgeAdj
Sensory	Contrast Sensitivity	Mars_Final
Personality	Openness	NEOFAC_O
Personality	Conscientiousness	NEOFAC_C
Personality	Extroversion	NEOFAC_E
Personality	Agreeableness	NEOFAC_A
Personality	Neuroticism	NEOFAC_N

References

1. Greene, A. S., Gao, S., Scheinost, D. & Constable, R. T. Task-induced brain state manipulation improves prediction of individual traits. *Nat. Commun.* **9**, 2807 (2018).
2. Barch, D. M. *et al.* Function in the human connectome: task-fMRI and individual differences in behavior. *Neuroimage* **80**, 169–189 (2013).
3. Rosenberg, M. D. *et al.* Behavioral and Neural Signatures of Working Memory in Childhood. *J. Neurosci.* **40**, 5090–5104 (2020).
4. Pessoa, L., Gutierrez, E., Bandettini, P. & Ungerleider, L. Neural correlates of visual working memory: fMRI amplitude predicts task performance. *Neuron* **35**, 975–987 (2002).
5. Spearman, C. The Proof and Measurement of Association between Two Things. *The American Journal of Psychology* vol. 15 72 (1904).
6. Iscan, Z. *et al.* Test-retest reliability of freesurfer measurements within and between sites: Effects of visual approval process. *Hum. Brain Mapp.* **36**, 3472–3485 (2015).
7. Noble, S. *et al.* Influences on the Test–Retest Reliability of Functional Connectivity MRI and its Relationship with Behavioral Utility. *Cerebral Cortex* vol. 27 5415–5429 (2017).
8. Zelazo, P. D. & Bauer, P. J. *National Institutes of Health Toolbox Cognition Battery (NIH Toolbox CB): Validation for Children Between 3 and 15 Years*. (Wiley-Blackwell, 2013).
9. Achenbach, T. M. & Ruffle, T. M. The Child Behavior Checklist and Related Forms for Assessing Behavioral/Emotional Problems and Competencies. *Pediatrics in Review* vol. 21 265–271 (2000).
10. Kragel, P. A., Han, X., Kraynak, T., Gianaros, P. J. & Wager, T. D. fMRI can be highly reliable, but it depends on what you measure. doi:10.31234/osf.io/9eaxk.
