# Supplementary material for: Reproducible brain-wide association studies require thousands of individuals
Source: Nature. 2022 Mar 16;603(7902):654–60. doi: 10.1038/s41586-022-04492-9 (PMC8991999; doi:10.1038/s41586-022-04492-9)
Supplement: Supplementary file 2 — Reporting Summary [file 41586_2022_4492_MOESM2_ESM.pdf]

nature portfolio

Corresponding author(s): Scott Marek, Brenden Tervo-Clemmens, Nico Dosenbach

Last updated by author(s): Jan 10, 2022

Reporting Summary

Nature Portfolio wishes to improve the reproducibility of the work that we publish. This form provides structure for consistency and transparency in reporting. For further information on Nature Portfolio policies, see our Editorial Policies and the Editorial Policy Checklist.

Statistics

For all statistical analyses, confirm that the following items are present in the figure legend, table legend, main text, or Methods section.

n/a	Confirmed	
☐	☒	The exact sample size (*n*) for each experimental group/condition, given as a discrete number and unit of measurement
☐	☒	A statement on whether measurements were taken from distinct samples or whether the same sample was measured repeatedly
☐	☒	The statistical test(s) used AND whether they are one- or two-sided<br>*Only common tests should be described solely by name; describe more complex techniques in the Methods section.*
☐	☒	A description of all covariates tested
☐	☒	A description of any assumptions or corrections, such as tests of normality and adjustment for multiple comparisons
☐	☒	A full description of the statistical parameters including central tendency (e.g. means) or other basic estimates (e.g. regression coefficient) AND variation (e.g. standard deviation) or associated estimates of uncertainty (e.g. confidence intervals)
☐	☒	For null hypothesis testing, the test statistic (e.g. *F*, *t*, *r*) with confidence intervals, effect sizes, degrees of freedom and *P* value noted<br>*Give P values as exact values whenever suitable.*
☒	☐	For Bayesian analysis, information on the choice of priors and Markov chain Monte Carlo settings
☒	☐	For hierarchical and complex designs, identification of the appropriate level for tests and full reporting of outcomes
☐	☒	Estimates of effect sizes (e.g. Cohen's *d*, Pearson's *r*), indicating how they were calculated

Our web collection on statistics for biologists contains articles on many of the points above.

Software and code

Policy information about availability of computer code

Data collection	No software was used for data collection. Neuroimaging and behavioral data were from existing, open-source datasets (ABCD, UKB, HCP) whose acquisition's are presented in detail in previous work. The ABCD Study data were collected between 2016-2018. The HCP data were collected between 2010-2016. The UKB data collection started in 2014 and 2015 to demonstrate the feasibility of high-throughput imaging and to finalize the imaging protocols required for the main phase. Funding was then released to extend the imaging enhancement to an additional 95,000 participants, with data collection estimated to finish by 2023.
Data analysis	MRI data analysis code can be found here: https://github.com/ABCD-STUDY/nda-abcd-collection-3165<br>ABCD and UKB MRI data processing code can be found here https://github.com/DCAN-Labs/abcd-hcp-pipeline<br>Manuscript analysis code can be found here https://gitlab.com/DosenbachGreene/bwas<br>FIRMM software: https://firmm.readthedocs.io/en/latest/release_notes/. ABCD uses version 3.0.14.<br>MuMln R package: https://cran.r-project.org/web/packages/MuMIn/index.html. Version 1.43.17.

For manuscripts utilizing custom algorithms or software that are central to the research but not yet described in published literature, software must be made available to editors and reviewers. We strongly encourage code deposition in a community repository (e.g. GitHub). See the Nature Portfolio guidelines for submitting code & software for further information.

Data

Policy information about availability of data

All manuscripts must include a data availability statement. This statement should provide the following information, where applicable:

- Accession codes, unique identifiers, or web links for publicly available datasets
- A description of any restrictions on data availability
- For clinical datasets or third party data, please ensure that the statement adheres to our policy

Participant level data from all datasets (ABCD, HCP, UKB) is openly available pursuant to individual, consortia-level data access rules. The ABCD data repository grows and changes over time. The ABCD data used in this report came from ABCD collection 3165 and the Annual Release 2.0, DOI 10.15154/1503209.
The UK Biobank is a large-scale biomedical database and research resource containing genetic, lifestyle and health information from half a million UK participants (www.ukbiobank.ac.uk). UK Biobank's database, which includes blood samples, heart and brain scans and genetic data of the 500,000 volunteer participants, is globally accessible to approved researchers who are undertaking health-related research that's in the public interest.
Data were provided, in part, by the Human Connectome Project, WU-Minn Consortium (Principal Investigators: David Van Essen and Kamil Ugurbil; 1U54MH091657) funded by the 16 NIH Institutes and Centers that support the NIH Blueprint for Neuroscience Research; and by the McDonnell Center for Systems Neuroscience at Washington University. Some data used in the present study are available for download from the Human Connectome Project (www.humanconnectome.org). Users must agree to data use terms for the HCP before being allowed access to the data and ConnectomeDB, details are provided at https://www.humanconnectome.org/study/hcp-young-adult/data-use-terms.
No new data were collected for this manuscript. Across the ABCD, HCP, and UKB, we downloaded data between 01/2019 - 10/2021. We did not use any specific software for downloading the data. For details on data collection in ABCD (baseline data), see Casey et al., 2018; in HCP (1200 release) see Van Essen et al., 2013; in UKB see Sudlow et al., 2015.

Field-specific reporting

Please select the one below that is the best fit for your research. If you are not sure, read the appropriate sections before making your selection.

☐ Life sciences ☒ Behavioural & social sciences ☐ Ecological, evolutionary & environmental sciences

For a reference copy of the document with all sections, see nature.com/documents/nr-reporting-summary-flat.pdf

Behavioural & social sciences study design

All studies must disclose on these points even when the disclosure is negative.

Study description

Quantitative analyses of the magnitude and reproducibility of cross-sectional associations between neuroimaging measures and psychological/psychiatric phenotypes.

Research sample

Because our main focus was to estimate the effect size of BWAS, which requires a very large sample, this project uses open-access data from three of the largest community-recruited neuroimaging samples (ABCD, HCP, UKB) that contain both structural and functional MRI data. Combined, the aggregate sample is one of the largest (N~50,000) and most representative neuroimaging samples, although none of the samples is fully population representative. See manuscript for extended discussion.

Sampling strategy

All samples were recruited from the community (ABCD & HCP from the USA and UK Biobank from the United Kingdom). Individual samples (ABCD, HCP, UKB) used unique sample size calculations and sampling strategies which are discussed in prior work with these open source datasets (Casey et al., 2018, Van Essen et al., 2013, and Littlejohns et al., 2020, respectively).

Data collection

All data were from existing data repositories and were downloaded between 01/2019 - 10/2021. Data used in the manuscript were from existing large consortia datasets (ABCD: see Casey et al., 2018 & Barch et al., 2018; HCP: We used data from the 1200 subjects data release (van Essen et al., 2013); The UKB brain imaging component has been described in Miller et al. (2016), and the processing and quality control described in Alfaro-Almagro et al. (2018)). Because we did not personally collect any of the data used in this manuscript, all data were from existing data repositories and researchers were therefore not blind to the source of the data.

Timing

ABCD: see Casey et al., 2018
HCP: see van Essen et al., 2013
UKB: see Miller et al., 2016

Data exclusions

In ABCD, we used strict inclusion criteria with regard to head motion. Specifically, inclusion criteria for the current project (see ref 30 in manuscript for broader ABCD inclusion criteria) consisted of at least 600 frames (8 minutes) of low-motion (filtered FD<0.08) resting state functional connectivity data. Our final dataset consisted of data from a total of N=3,928 youth across the discovery (N=1,964) and replication (N=1,964) sets. The final discovery and replication sets did not differ in mean FD ($\Delta M=0.002$, $t=0.60$, $p=0.55$) or total frames included ($\Delta M=6.4$, $t=0.94$, $p=0.35$). The subject lists for ARMS samples and our associated matrices will be released in the ABCD-BIDS Community Collection (ABCD collection 3165) for community use.
For HCP data, we used similar data quantity inclusion, as well as an FD < 0.20 (unfiltered FD). This resulted in the inclusion of N=900 individuals (N=877 across all NIH Toolbox subscales).
Given the UK Biobank data only contains 6 mins of resting state data, we did not exclude any subjects due to low quantities of data post motion censoring (FD < 0.08, filtered).

Non-participation

N/A

Randomization	All three samples were observational studies and no randomization was used.

Reporting for specific materials, systems and methods

We require information from authors about some types of materials, experimental systems and methods used in many studies. Here, indicate whether each material, system or method listed is relevant to your study. If you are not sure if a list item applies to your research, read the appropriate section before selecting a response.

Materials & experimental systems

n/a	Involved in the study
☒	☐ Antibodies
☒	☐ Eukaryotic cell lines
☒	☐ Palaeontology and archaeology
☒	☐ Animals and other organisms
☐	☒ Human research participants
☒	☐ Clinical data
☒	☐ Dual use research of concern

Methods

n/a	Involved in the study
☒	☐ ChIP-seq
☒	☐ Flow cytometry
☐	☒ MRI-based neuroimaging

Human research participants

Policy information about studies involving human research participants

Population characteristics	See above.
Recruitment	*Describe how participants were recruited. Outline any potential self-selection bias or other biases that may be present and how these are likely to impact results.*
Ethics oversight	The ABCD Study obtained centralized institutional review board approval from the University of California, San Diego, and each of the 21 study sites obtained local institutional review board approval. Ethical regulations were followed during data collection and analysis. Parents or caregivers provided written informed consent, and children gave written assent.

Note that full information on the approval of the study protocol must also be provided in the manuscript.

Magnetic resonance imaging

Experimental design

Design type	resting-state fMRI, task-based fMRI; structural (cortical thickness) MRI
Design specifications	ABCD resting state: 4, 5 min runs, eyes open<br>HCP resting state: 4, 15 min runs, eyes open<br>UKB resting state: 1, 6 min run, eyes open
Behavioral performance measures	Primary analyses use cognitive assessments from the NIH Toolbox and psychopathology assessment Child Behavior Checklist (see manuscript for individual subscales, total of 41) included in standard data releases and discussed in detail perviously (Barch et al., 2018)

Acquisition

Imaging type(s)	Resting-state fMRI, task-fMRI, structural (cortical thickness) MRI
Field strength	3 Tesla
Sequence & imaging parameters	Primary analyses use open-source distributed fMRI and MR data that adhere to consortia guidelines (see Casey et al., 2018, Van Essen et al., 2013, and Miller et al., 2016 for ABCD, HCP, and UKB parameters, respectively).
Area of acquisition	Whole brain
Diffusion MRI	☐ Used ☒ Not used

Preprocessing

Preprocessing software	Preprocessing of ABCD was done using a suite of tools. All code can be found here: https://github.com/ABCD-STUDY/nda-abcd-collection-3165. Individual datasets (ABCD, UKB, HCP) and individual study sites (e.g., ABCD site 1 versus site 2) used unique sequence and imaging parameters which are discussed in prior work introducing these open-source datasets.

Normalization	1) PreFreesurfer normalizes anatomical data. This normalization entails brain extraction, denoising, and then bias field correction on anatomical T1 and/or T2 weighted data. The ABCD-HCP pipeline includes two additional modifications to improve output image quality. ANTs 65 DenoiseImage models scanner noise as a Rician distribution and attempts to remove such noise from the T1 and T2 anatomical images. Additionally, ANTs N4BiasFieldCorrection attempts to smooth relative image histograms in different parts of the brain and improves bias field correction. 2) FreeSurfer 1 constructs cortical surfaces from the normalized anatomical data. This stage performs anatomical segmentation, white/grey and grey/CSF cortical surface construction, and surface registration to a standard surface template. Surfaces are refined using the T2 weighted anatomical data. Mid-thickness surfaces, which represent the average of white/grey and grey/CSF surfaces, are generated here. 3) PostFreesurfer converts prior outputs into an HCP-compatible format (i.e. CIFTIs) and transforms the volumes to a standard volume template space using ANTs nonlinear registration, and the surfaces to the standard surface space via spherical registration.
Normalization template	The "Vol" stage corrects for functional distortions via reverse-phase encoding spin-echo images. All resting state runs underwent intensity normalization to a whole brain mode value of 1000, within run correction for head movement, and functional data registration to the standard template (MNI). Atlas transformation was computed by registering the mean intensity image from each BOLD session to the high resolution T1 image, and then applying the anatomical registration to the BOLD image. This atlas transformation, mean field distortion correction, and resampling to 3-mm isotropic atlas space were combined into a single interpolation using FSL's 66 applywarp tool. The "Surf" stage projects the normalized functional data onto the template surfaces.
Noise and artifact removal	Additional BOLD preprocessing steps were executed to reduce spurious variance unlikely to reflect neuronal activity 46. First, a respiratory filter was used to improve FD estimates calculated in the volume ("vol") stage68. Second, temporal masks were created to flag motion-contaminated frames using the improved FD estimates 63. Frames with a filtered FD>0.3mm were flagged as motion-contaminated for nuisance regression only. After computing the temporal masks for high motion frame censoring, the data were processed with the following steps: (i) demeaning and detrending, (ii) interpolation across censored frames using least squares spectral estimation of the values at censored frames so that continuous data can be (iii) denoised via a GLM with whole brain, ventricular, and white matter signal regressors, as well as their derivatives. Denoised data were then passed through (iv) a band-pass filter (0.008 Hz<f<0.10 Hz) without re-introducing nuisance signals 69 or contaminating frames near high motion frames.
Volume censoring	Yes, ABCD data were censored at a filtered frame-wise displacement of < 0.08mm and HCP data were filtered using a non-filtered framewise displacement of <0.20mm.

Statistical modeling & inference

Model type and settings	Mass univariate and multivariate (support vector regression, canonical correlation analysis). Multiple parameterizations of each of these models were explored with the stated goal being to determine field-wide reproducibility in brain-phenotype association studies (see manuscript).
Effect(s) tested	As the primary aim of the paper was to determine the general reproducibility of brain-phenotype effects, multiple scales and combinations of effects were examined. Owing to the cross-sectional, nature of these studies, all effects are between-person associations.

Specify type of analysis: ☐ Whole brain ☐ ROI-based ☒ Both

Anatomical location(s)	Parcel-level and network-level analyses utilized the field-standard Gordon et al., 2016, Cerebral Cortex, and Seitzman et al., 2020, NeuroImage. Vertex-wise and voxel-wise data were extracted from Ciftis.
Statistic type for inference (See Eklund et al. 2016)	Multiple levels of neuroanatomical scale were used, including voxels, regions of interest, and networks.
Correction	As the primary aim of the paper was to determine the general reproducibility of brain-phenotype effects, multiple levels of significance values and correction were used, ranging from uncorrected to bonferroni (FWER) correction.

Models & analysis

n/a	Involved in the study
☐	☒ Functional and/or effective connectivity
☒	☐ Graph analysis
☐	☒ Multivariate modeling or predictive analysis

Functional and/or effective connectivity	Pearson correlation
Multivariate modeling and predictive analysis	Multivariate Out-of-Sample Replication: Support Vector Regression (SVR)<br>Support vector regression (SVR) with a linear kernel was performed using the e1071 package in the R environment (version 3.5.2) to predict primary phenotypes (psychopathology, cognitive ability) and other demographics and psychological phenotypes from individual differences in either RSFC or cortical thickness. One hundred bootstrap samples (sampling with replacement) were generated for each sample size. Hyperparameter tuning was examined in 1) split halves of the full discovery sample for multiple cognitive (NIH Toolbox) and psychopathology (CBCL) symptoms and 2) 10-fold cross-validation within the full discovery sample for primary phenotypes (psychopathology, cognitive ability), but did not appreciably change out-of-sample prediction estimates to the replication sample (e.g., average out-of-sample correlation difference between tuned and non-tuned models : RSFC = -0.006, Cortical Thickness = 0.014). Fig. 4A,C use default

hyperparameters and PCA dimensionality reduction (with a threshold of 50% variance explained in the discovery set, for each sample size) prior to SVR, given that this procedure balanced out-of-sample prediction and model complexity for nearly all model types. Replication set data were not used to estimate principal components, but rather replication set data were projected into component space via independently estimated loading matrices for each subsample of the discovery set to prevent bias. An alternative strategy of univariate feature ranking was also examined, where SVR models were trained on the 5,000, 10,000, or 15,000 vertices (cortical thickness) or edges (RSFC) with the highest correlation to the variable of interest in the training dataset, but this approach resulted in lower out-of-sample prediction. Significance thresholds for out-of-sample replication were estimated via permutation testing (1,000 iterations) with models trained on the full discovery set (RSFC: N=1964; cortical thickness: N=1,814) and tested on the full replication set.

Multivariate Out-of-Sample Replication: Canonical Correlation Analysis (CCA)
Canonical correlation analysis (CCA) was performed using Matlab's (2019b) cannoncor.m function to predict the NIH Toolbox and CBCL from individual differences in either RSFC or cortical thickness. Equivalent bootstrapping and subsampling of the discovery set were tested and applied to the replication set, as in the SVR analyses. In order to model sampling variability across sample sizes, 100 bootstrap (sampling with replacement) samples were generated for each sample size. As with SVR, Fig. 4B,D used principal-component analysis (PCA) dimensionality reduction (threshold of 20% variance explained in the discovery set, for each sample size) prior to CCA given that this maximised out-of-sample prediction. CCA models were fit on iteratively larger subsamples of the discovery (in-sample) data set. The first canonical vector was extracted and applied to the full replication (out-of-sample) brain data to predict replication set (out-of-sample) behavioral phenotype. Prediction accuracy was quantified by expressing the correlation between the matrix products of the first canonical vector (from the discovery set) and replication brain and phenotypic data. Significance thresholds for out-of-sample replication were estimated via permutation testing (1,000 iterations) with models trained on the full discovery set (RSFC: N=1964; cortical thickness: N=1,814) and tested on the full replication set.
